# Spontaneous idiopathic intratesticular hemorrhage as a differential diagnosis for acute scrotal pain

**DOI:** 10.1186/s12894-023-01255-0

**Published:** 2023-05-03

**Authors:** Saleh Abuorouq, Husam K. Haddad, Mohannad Alomari, Tawfeeq El-Far, Hiba Alzoubi, Mohammad Alqudah, Hashem Abu Serhan

**Affiliations:** 1 Urology Division, Department of Clinical Medical Sciences, Faculty of Medicine, Yarmouk University, Irbid, Jordan; 2 Department of Pathology and Laboratory Medicine, Ministry of Health, Amman, Jordan; 3 Private Sector, Irbid, Jordan; 4 Department of Basic Medical Sciences, Faculty of Medicine, Yarmouk University, Irbid, Jordan; 5 Department of Pathology and Microbiology, Faculty of Medicine, Jordan University of Science and Technology, Irbid, Jordan; 6 Department of Pathology, Microbiology and Forensic Medicine, Faculty of Medicine, Hashemite University, Zarqa, Jordan; 7 Department of Ophthalmology, Hamad Medical Corporations, PO Box: 3050, Doha, Qatar

**Keywords:** Acute scrotal pain, Hemorrhage, Orchiectomy, Testicles, Emergency

## Abstract

**Background:**

Spontaneous idiopathic testicular hemorrhage is an extremely rare entity with few published reports in the literature.

**Case presentation:**

We report a case of a 15-year-old boy who had been experiencing intense, left scrotal pain for the previous twelve hours. No previous history of trauma or bleeding disorders. The left testis was enlarged and tender. Left orchiectomy was performed. The entire testis was dusty and dark grossly. Microscopic sections show diffuse intratesticular bleeding with intact seminiferous tubules and spermatogenesis.

**Conclusions:**

Spontaneous idiopathic testicular hemorrhage should be considered when evaluating patients with acute scrotal pain. Clinical and ultrasonographic findings and histopathologic evaluation are mandatory to diagnose it.

## Introduction

Acute scrotal pain is one of the urological emergencies that can be attributed to different causes and leads to testicular hemorrhage. The differential diagnosis of testicular hemorrhage is wide including trauma, torsion, infection, coagulopathy, tumors, or, rarely, vasculitis. One of the rare causes of testicular bleeding that should be also taken into consideration is idiopathic spontaneous intratesticular hemorrhage which has no identifiable risk factors [1–5]. This entity was first described in 1914.

## Case presentation

A previously healthy 15-year-old boy presented with acute-onset left testicular pain for 12 h. He was afebrile, denied having any dysuria or hematuria, and denied any history of previous trauma. Physical examination showed a left testicle that was diffusely enlarged without any discrete masses, On palpation, the left testis was tender and swollen. The right testis was normal. Ultrasonography shows heterogeneous enlargement of the left testis with areas of decreased echogenicity. No associated masses are identified. Testicular doppler ultrasonography shows absent blood flow. Surgical exploration was decided urgently to save the testicles.

**Fig. 1:**
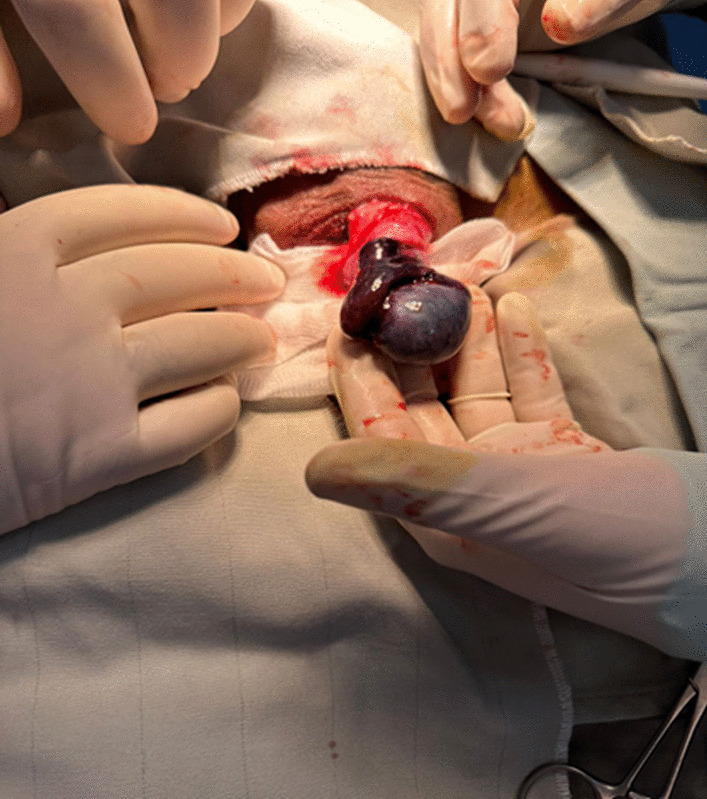
Perioperative image of the testis showed uniformly enlarged and diffuse dusty dark discoloration of the left testis and epididymis without any associated masses

**Fig. 2:**
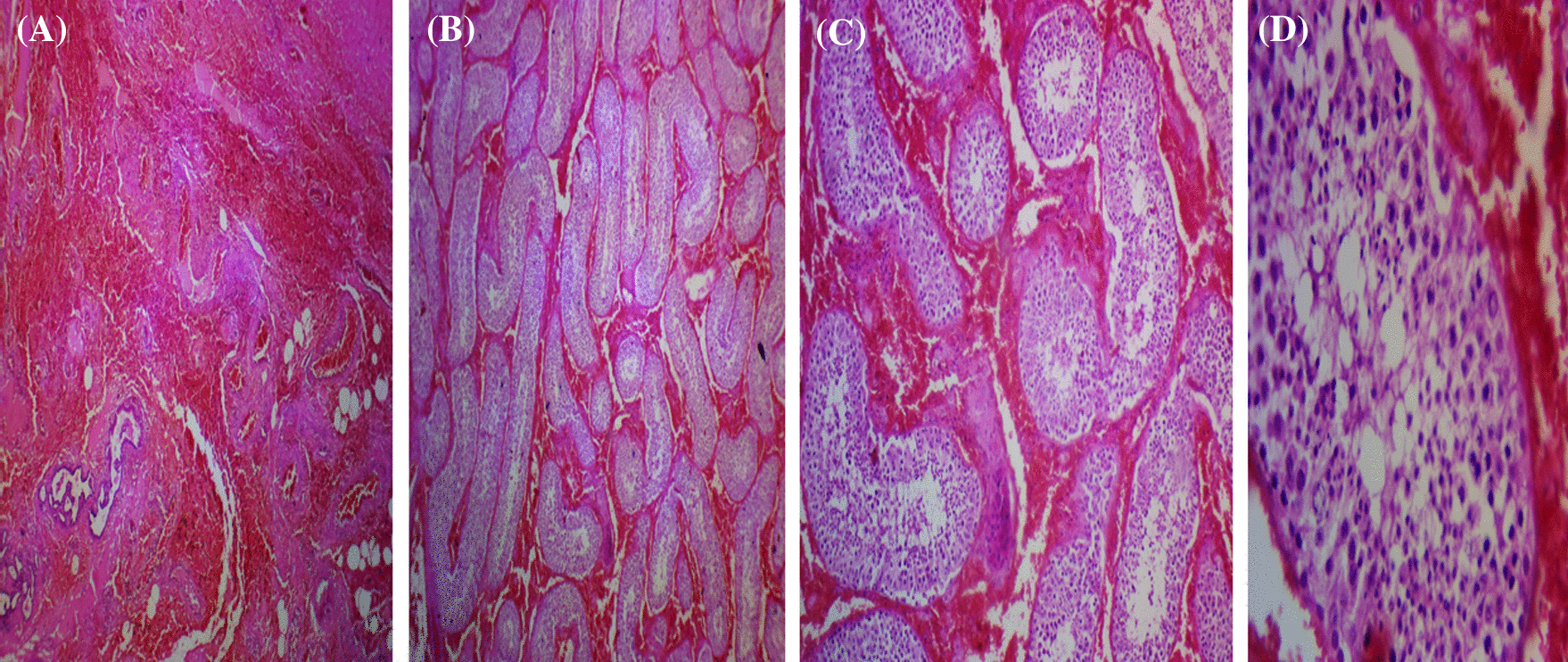
(**A**, **B**, **C**, and **D**), (10 × , 20 × , and 40 ×) showed diffuse testicular interstitial hemorrhage with intact seminiferous tubules and full spermatogenesis

Upon surgical exploration, the whole testis and epididymis were diffusely enlarged and dusty dark (Fig. 1), consequently, unilateral left orchiectomy was performed. The Microscopic sections show diffuse interstitial hemorrhage with intact seminiferous tubules and full spermatogenesis (Fig. 2A–D). There was no sign of thrombosis, vasculitis, tumors, tumor necrosis, or any other pathologies that could be concurrently present or lead to testicular bleeding.

## Discussion

Idiopathic intratesticular hemorrhage is a rare disease that was first described in 1914 [1]. Only a few cases have been reported in the literature [1–5]. Most patients present with acute onset of unilateral scrotal pain associated with swelling [1–5]. The differential diagnosis for this presentation is wide and includes epididymitis, torsion, trauma, or testicular tumors, and this rare entity should also be taken into consideration.

Scrotal sonography is the gold standard imaging modality for evaluating patients with testicular pain and swelling, especially since it is fast, simple, and noninvasive. It helps in determining the nature of the swelling, and whether it is solid or cystic [3]. The addition of color Doppler imaging aids in the identification of the lesion’s vascularity [3]. In the present case, the distinction between intratesticular hemorrhage and testicular torsion was not possible, especially since color Doppler ultrasonography shows absent blood flow. Despite the fact that sonography is the gold standard for diagnosing intratesticular masses, there are no pathognomic findings for this rare entity as reported in different cases reported in the literature [2–5]. An echogenic solid or cystic mass with no internal color Doppler flow signals is the most likely appearance [3–5]. Absence of blood flow is caused by this spontaneous hemorrhage that lead to compression of blood vessels, moreover, it is one of the signs that make the distinction between this entity and testicular torsion very difficult and challenging [5].

Magnetic resonance imaging (MRI) may be used to evaluate an acute scrotum when the results of color Doppler are equivocal. Intratesticular hematomas appear as a homogeneous mass with a moderate to intermediate signal intensity on T1- and T2-weighted sequences. To exclude the possibility of malignant tumors, a dynamic MRI contrast enhancement approach can be applied to distinguish the intratesticular hemorrhage that can be treated conservatively from tumors that require surgery since malignant tumors exhibit contrast enhancement with good diagnostic accuracy [3].

The gross features, absence of blood flow and the black discoloration of the testis in idiopathic intratesticular hemorrhage could resemble the necrotic testicle as in our case, and mandates its removal. However, one of the cases reported the normal color of the involved spermatic cord, which would lessen the likelihood of testicular torsion [1]. Additionally, this entity could mimic testicular neoplasm [2, 3], because malignancy cannot be ruled out at an early stage of diagnosis, the majority of patients and healthcare professionals choose surgical exploration with a potential orchiectomy.

The final diagnosis for most of the cases was made only after surgical exploration and unilateral orchiectomy [1, 3, 4], but the best course of action might be to quickly remove the affected tissue for a frozen section in order to potentially save the testicle [2, 5]. This is decisive but invasive as well, and it may be prevented if the diagnosis of spontaneous intratesticular hemorrhage is determined with certainty by other diagnostic modalities. One of the reported cases in the literature discussed the first testicular salvage in an adult with idiopathic spontaneous testicular hemorrhage based on sonographic and perioperative findings, accordingly, the testicle was preserved [2]. Additionally, there is a similar first case report in the pediatric population [5]. Accordingly, the accurate diagnosis of a spontaneous intratesticular hemorrhage depends on many factors including the physician’s having a high degree of suspicion for this rare entity, in addition to the clinical history and sonographic findings. MRI can be crucial in making the diagnosis of spontaneous intratesticular hemorrhage when sonographic results are inconclusive. It is very crucial to rule out bleeding disorders, predisposing factors, and trauma before the diagnosis of spontaneous intratesticular hemorrhage.

## Conclusion

We reported a case of idiopathic spontaneous intratesticular hemorrhage which is a rare entity. It is still challenging to diagnose this entity and decide how to save the testis if this is possible. This rare entity should be included in the differential diagnosis of unilateral acute scrotal pain. Clinical, radiologic, and histopathological correlation are mandatory to reach the diagnosis.

## Data Availability

All data generated or analyzed during this study are included in this published article.
